# Fatty Acid Uptake in T Cell Subsets Using a Quantum Dot Fatty Acid Conjugate

**DOI:** 10.1038/s41598-017-05556-x

**Published:** 2017-07-19

**Authors:** Megan E. Muroski, Jason Miska, Alan L. Chang, Peng Zhang, Aida Rashidi, Haley Moore, Aurora Lopez-Rosas, Yu Han, Maciej S. Lesniak

**Affiliations:** 1 Department of Neurological Surgery, Feinberg School of Medicine, Northwestern University, Chicago, USA; 2 Committee on Cancer Biology, The University of Chicago, Chicago, USA

## Abstract

Fatty acid (FA) metabolism directly influences the functional capabilities of T cells in tumor microenvironments. Thus, developing tools to interrogate FA-uptake by T cell subsets is important for understanding tumor immunosuppression. Herein, we have generated a novel FA-Qdot 605 dye conjugate with superior sensitivity and flexibility to any of the previously commercially available alternatives. For the first time, we demonstrate that this nanoparticle can be used as a specific measure of fatty acid uptake by T cells both *in-vitro* and *in-vivo*. Flow cytometric analysis shows that both the location and activation status of T cells determines their FA uptake. Additionally, CD4+ Foxp3+ regulatory T cells (Tregs) uptake FA at a higher rate than effector T cell subsets, supporting the role of FA metabolism for Treg function. Furthermore, we are able to simultaneously detect glucose and fatty acid uptake directly within the tumor microenvironment. Cumulatively, our results suggest that this novel fluorescent probe is a powerful tool to understand FA utilization within the tumor, thereby providing an unprecedented opportunity to study T cell FA metabolism *in-vivo*.

## Introduction

While immunotherapy has shown incredible promise as a tool for cancer therapy^1–3^, many patients are not responsive to these treatments. One emerging explanation is that the metabolic requirements for effector immune cells to proliferate and ultimately destroy malignant tissue is not met within the tumor microenvironment (TME)^4–6^. T The heightened glycolytic metabolism of tumors potently inhibits the functionality of effector T cells through extracellular acidification^7^ and nutrient competition^6^. Importantly, inhibition of these processes is thought to lead to productive anti-tumor immune responses^7, 8^. While these studies have provided integral insight into how effector T cells function under the metabolic influence of the tumor, there is no information on how regulatory T cells (Tregs) function in these circumstances.

Tregs are a potently immunosuppressive subset of CD4+ T cells expressing the transcription factor Foxp3^9^, which accumulates in most solid tumors^10^ including glioblastoma multiforme^11–13^. In order for Tregs to thrive under the same glycolytic constraints that inhibit effector T cells, an alternative bioenergetic pathway is needed. Previous studies have suggested that Tregs preferentially rely on fatty acid oxidation (FAO)^14^ and obtain fatty acids exogenously^15^. Although promising, there is no evidence that this metabolic pathway is used by Tregs to thrive within the tumor microenvironment. Therefore, the ability to monitor the metabolic phenotypes of these cells *in-vivo* may be critical to our understanding of how immunosuppression is propagated in the tumor.

Furthermore, understanding the distinct methods by which T cells uptake nutrients on a single cell basis will be informative for understanding how these cells persist. Previously, the only method to simultaneously measure glucose and fatty acid uptake is based on fluorescent dyes with almost identical spectral characteristics^16, 17^. This severely hampers the ability to understand the inherent complexity and heterogeneous nature of T cells. Some T cells may utilize one pathway over another, which has been suggested by previous studies demonstrating that fatty acid uptake inhibits glucose uptake, and vice-versa^18, 19^. The ability to measure exogenous metabolite uptake provides researchers with the ability to determine how the cells are utilizing energy from the microenvironment. Fatty acid uptake is coordinated with metabolic functions of the cell, and within T cells, plays an integral part in differentiation^20^. Activated T cells preferentially utilize aerobic glycolysis to fuel the biosynthesis of new proteins, lipid, and nucleic acids for cellular proliferation, whereas memory or Tregs prefer to pick up free fatty acids and oxidize them to provide ATP, Acetyl-CoA, and NADPH for long term survival in tissues^21^. Determining cellular energy utilization within distinct cell subsets provides researchers with potential strategies for future cancer and immunotherapy applications.

To address these questions, we have developed a sensor for fatty acid uptake using fatty acids conjugated to the surface of a quantum dot. We demonstrate that this sensor is more sensitive than the current dye-based approaches and is sensitive enough to be detected for *in-vivo* applications. The wide array of quantum dots available and the flexibility of its thiol chemistry makes this platform a versatile tool that can be modified in both color and lipid composition for many future applications. Herein, we demonstrate the ability to both append multiple lengths of FA to quantum dots and to append FA to broad-spectrum color quantum dots. This versatility allowed us to address the relative contribution of fatty acid uptake versus glucose uptake by T cells *in-vivo*, which, to our knowledge, is the first time this has been achieved.

## Results

### Verification of assembly of fatty acid conjugated quantum dots

**Figure 1:**
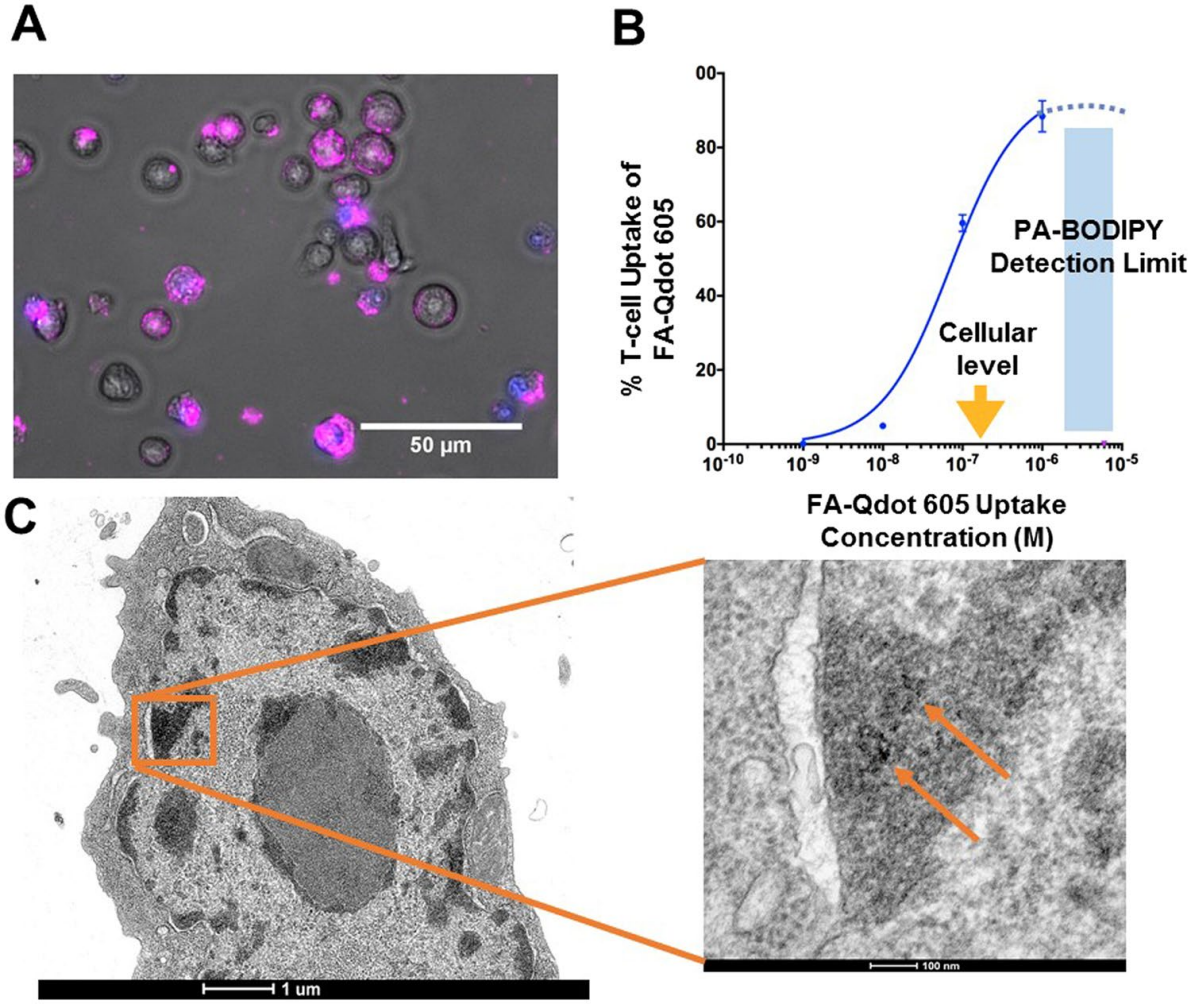
FA-Qdot 605 is taken up by activated T-cells. In (**A**), Epifluorescence imaging of T-cells in-vitro after incubation with FA-Qdot-605 conjugate (Purple), DAPI was added to solution to exclude cells undergoing apoptosis or those which have leaky membranes. In (**B**), Measurement of fatty acid Qdot-605 uptake at increasing concentrations as it compares to current FA-uptake detection via BODIPY dye. It is important to note that BODIPY uptake is less sensitive than cellular levels of palmitic acid (~200nM, yellow arrow). In (**C**), TEM image of FA-Qdot within a T-cell, image scale bars 1um and 100nm respectively, (4800x and 49000x zoom).

The Qdots are resuspended in chloroform and allowed to exchange with the aqueous FA, mercaptohexdecanoic acid, C16, at 60 °C in reducing conditions to form disulfide bonds with the sulfur on the ZnS surface. The resulting FA-Qdots are removed from excess FA before use and placed in PBS with 0.01% BSA. The differences in zeta potential between the FA-Qdot and the PEG-Qdot were −14.47 and +3.94, as shown in Supplemental Fig. 1A. This data suggests that the FA-Qdot uptake is specific to the FA on the Qdot and not due to the surface charge, as a negative surface charge does not readily attract the Qdot to the cell surface. In addition, we quantified the amount of FA on the surface of the Qdot through the use of a Free Fatty Acid assay and determined that our FA-Qdots contain 244 ± 5.9 FA per Qdot Supplemental Fig. 1B. To verify the ability of T cells to uptake the FA-Qdot conjugate, we pre-activated T cells and cultured them in the presence of the FA-Qdot conjugate for 3 minutes, washed, and monitored uptake via epifluorescence microscopy in Fig. 1A. Importantly, we also included a cell-impermeant DAPI dye and verified that FA-Qdot uptake was specific to living cells and not due to non-specific uptake by dead T cells. To quantify the sensitivity of our FA-Qdot conjugate, we performed a cellular uptake experiment using a concentration range of FA-Qdot (from 1 µM to 1 nM). Here, we observed that our conjugate was significantly more sensitive than standard PA-BODIPY in terms of detection limit. Furthermore, the total intracellular fatty acid calculated from our assays were comparable to hypothesized intracellular amounts, as shown in Fig. 1B. Finally, TEM of T cells demonstrate that the Qdots are able to effectively enter into the cells as shown in Fig. 1C.

### Fatty acid-conjugated quantum dots can measure fatty acid uptake in lymphocytes

**Figure 2:**
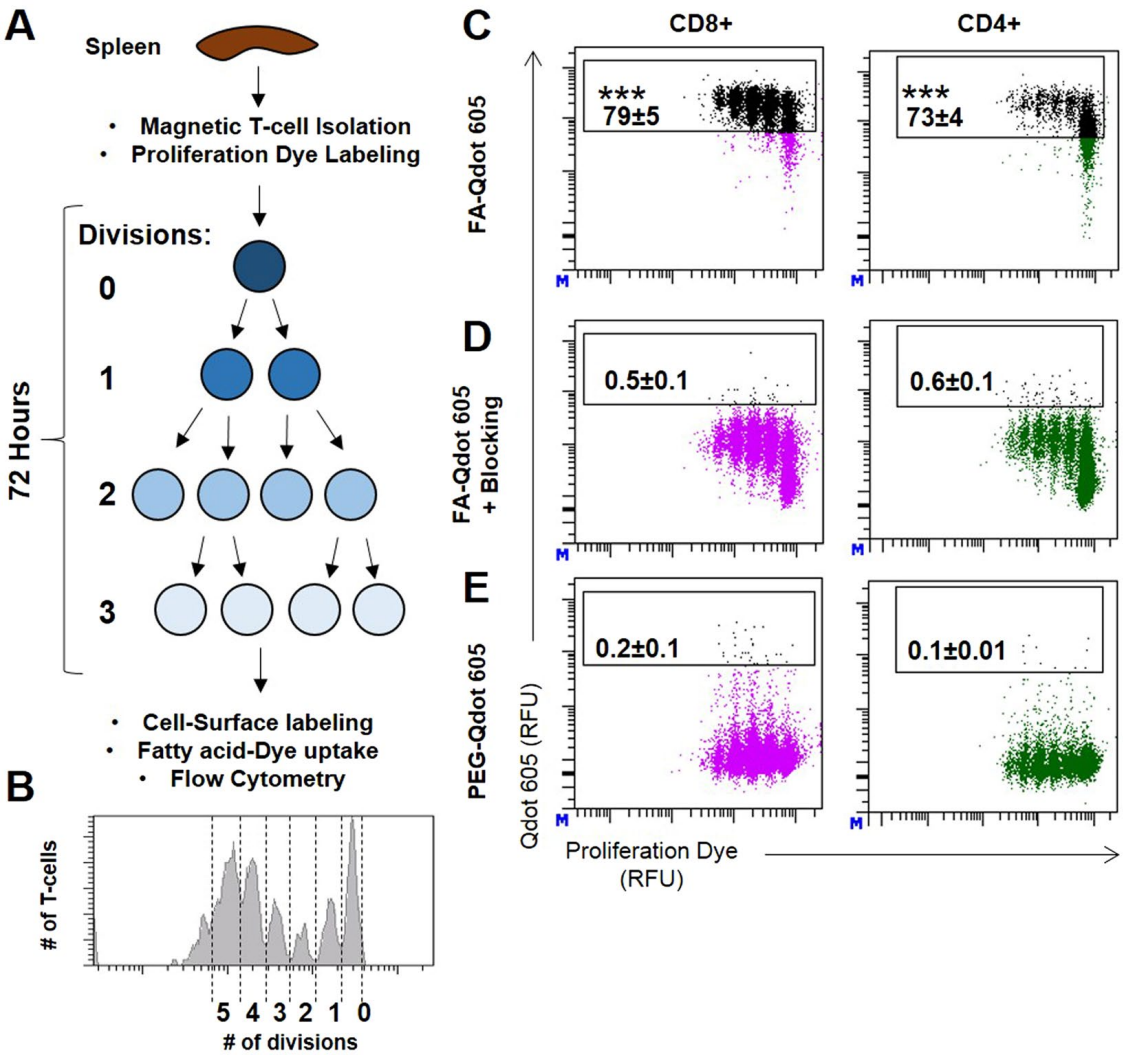
FA-Qdot 605 can accurately determine uptake by proliferating murine lymphocytes in culture. In (**A**), A schematic depicting isolation and labeling of T-cells with viability dye, followed by the steps before flow cytometric analysis. Importantly, after dye labeling, a cell will lose ½ of the dye each division, resulting in distinct peaks indicating cycles of proliferation. This parameter is normally shown as a histogram (**B**); however, with the inclusion of our FA-Qdot, we can compare the number of cellular divisions (X-axis) to amount of Fatty Acid uptake (y-axis) (**C**–**E**). In (**C**–**E**), Cell Trace Violet (CTV)-labeled T-cells were cultured under stimulating conditions of anti-CD3/anti-CD28 treatment for 72 hours and assayed for FA uptake utilizing flow cytometric analysis. In (**C**), stimulated T-cells were stained for T-cell markers and then incubated with 10nM of FA-Qdot 605 conjugate for 3 minutes before running flow analysis. In (**D**), to confirm specific uptake, stimulated T-cells were pre-incubated with 10X unlabeled palmitic acid for 10 minutes before staining with FA-Qdot 605 conjugate as in C. Amine-bound Qdot 605 was used in (**E**) to stain T-cells to test for non-specific uptake. Results are compiled from four separate experimental wells per group and is representative of 2 experiments. Statistics calculated as a percentage of the positive population ± SEM. One-way ANOVA, followed by Tukey’s post-hoc analysis, was used to calculate significance. p<0.05*; p<0.01**; p<0.001***. Relative Fluorescence Units = (RFU).

We wanted to verify that the FA-Qdot uptake was a product of the FA on the surface of the particle and not due to non-specific interactions of the Qdot. To determine whether FA-Qdot can accurately determine fatty acid uptake within lymphocytes, we harvested T cells from mouse spleens, stained with a proliferation dye, and then incubated for 72 hrs under T cell stimulating conditions. After stimulation and proliferation, T cells were then stained with viability dye and antibodies for flow cytometry, incubated with FA-Qdot, and subsequently analyzed via flow cytometry (Fig. 2). FA-Qdot conjugates were efficiently taken up by all activated T cells *in-vitro*, with the highest uptake observed in T cells that had undergone the most cell divisions. A schematic of the T cell labeling and cellular proliferation assay is demonstrated in Fig. 2A,B. In Fig. 2C–E, blocking studies were performed to determine that the route of uptake are the same compared to free FA. These studies ensure that the cell is saturated by free FA, and any uptake after blocking would suggest other routes of energy driven uptake, such as endocytosis. The assay was completed by incubating the cells with an excess of Palmitic acid prior to FA-Qdot staining, as previously demonstrated, to block specific uptake by a direct competitive inhibition^22^. Without any blocking, T cells incubated with 10 nM FA-Qdot resulted in 79.0% ± 5.0% and 73.0% ± 4.0% uptake within the CD8+ and CD4+ populations (Fig. 2C **)**. However, pre-incubation with sodium-palmitate added at a 1 mM concentration prior to adding FA-Qdot resulted in almost complete abrogation of FA-QDot uptake within the CD8+ and CD4+ populations (0.5% ± 0.1%, and 0.6% ± 0.1%, respectively) (Fig. 2D). Finally, to confirm that the FA-Qdot did not result in non-specific association with the surface, we stained the cells with Qdots that were coated with PEG, which resulted in minimal uptake compared to the blocking assay, with 0.2% ± 0.1% and 0.1% ± 0.01% uptake within the CD8+ and CD4+ populations, respectively (Fig. 2E). Furthermore, we demonstrate that we are able to get uptake with Qdots of different wavelengths, therefore expanding the versatility of this sensor. As shown in Supplemental Fig. 2, FA-Qdot with an emission of 660 nm had 94.0% ± 0.3% and 77.0% ± 0.6% uptake within the CD4+ and CD8+ populations, respectively, as compared to the Qdot with an emission of 605 nm. Furthermore, this strategy can be used with Qdots of different wavelengths/sizes, making this an easy method for determining FA uptake.

### Establishing differences of fatty acid uptake within T cell subsets during proliferation

**Figure 3:**
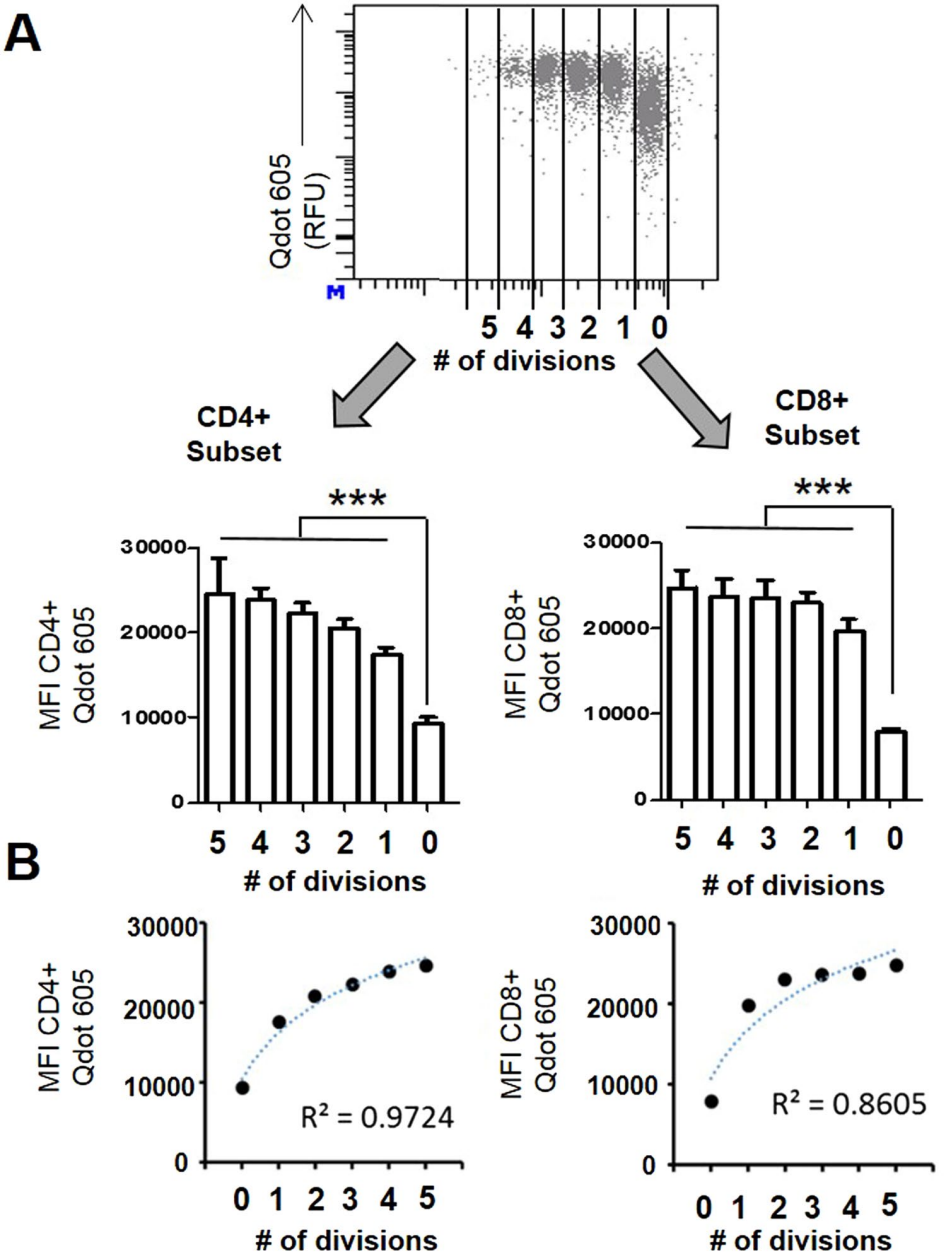
FA uptake by both T-cell subsets is positively correlated to the T-cell proliferation. Cell Trace Violet (CTV)-labeled T-cells were cultured under stimulating conditions of anti-CD3/anti-CD28 treatment for 72 hours and assayed for FA uptake utilizing flow cytometric analysis and compared against T-cell proliferation. The top panel (**A**) describes how T-cell proliferation is measured using this analysis; the bottom panels compare fatty acid uptake across different extents of proliferation in both CD4+ and CD8+ T-cell subsets. In (**B**), the average FA-Qdot 605 fluorescence for each cycle of proliferation was plotted, and a logarithmic regression was performed to determine the correlation between numbers of T-cell division and palmitic acid uptake. Results are complied from four separate experimental wells per group and are representative of 2 experiments. Statistics are calculated as the percentage of positive population ± SEM. One-way ANOVA, followed by Tukey’s post-hoc analysis, was used to calculate significance. p<0.05*; p<0.01**; p<0.001***. Relative Fluorescence Units = (RFU).

We further demonstrate that the Qdot is more sensitive than PA-BODIPY in determining FA uptake by observing the differences in uptake at 1 µM and 10 nM, as shown in Supplemental Fig. 3. As shown, 1 µM PA-BODIPY and 10 nM FA-Qdot have similar uptake in both CD4+ and CD8+ populations. 10 nM PA-BODIPY was not sensitive enough to detect differences under *in-vitro* conditions. This demonstrates that we are able to use 100x less FA-Qdot in determining FA uptake. We next wanted to verify that we could determine differences in proliferating populations.To verify that FA-Qdot conjugates were positively correlated within T cell proliferation, we performed a proliferation assay in which we stain T cells with the proliferation dye, Cell trace violet (CTV), a non-toxic dye that measures the number of times a T cell has undergone division within an allotted time^21^. CTV signal halves with every division, and we are able to accurately determine proliferating populations within T cells. We cultured T cells under stimulating conditions for 72 hrs and then measured the amount of CTV staining relative to the amount of FA-Qdot uptake. The cells were stained with FA-Qdot for 3 min, washed, and analyzed at the end of the 72hr period in order to directly quantify the amount of FA-Qdot uptake under differing levels of T cell proliferation (Fig. 3). In Fig. 3A, we show that the mean fluorescent intensity (MFI) with proliferating cells, all cells that have undergone 2+ divisions within 72 hrs have statistically significant uptake compared to the T cells that have not divided during this time. This suggests that actively proliferating cells are more likely to utilize exogenous FA, as compared to non-proliferating or inactive T cells. In addition, it does not appear that cells that have undergone more divisions take up more FA compared to cells that have only divided a few times, suggesting that T cells undergo a metabolic switch once they are activated. Furthermore, we show a positive correlation between more active subsets of T cells, as shown in Fig. 3B; logarithmic regression of the MFI resulted in R-squared values of 0.97 and 0.86 with CD4+ and CD8+ cells. These data suggest that the extent of T cell proliferation correlates with FA uptake and that non dividing cells are utilizing less fatty acids from the microenvironment. The differences in the CD4+ and CD8+ populations are consistent with previous studies suggesting Tregs preferentially rely on FA-uptake and FAO^23^. This demonstrates that the FA-Qdots are able to measure FA uptake in Treg cell populations and is a viable method *in-vitro*.

### Determination of fatty acid uptake of T cell subsets within the tumor microenvironment

**Figure 4:**
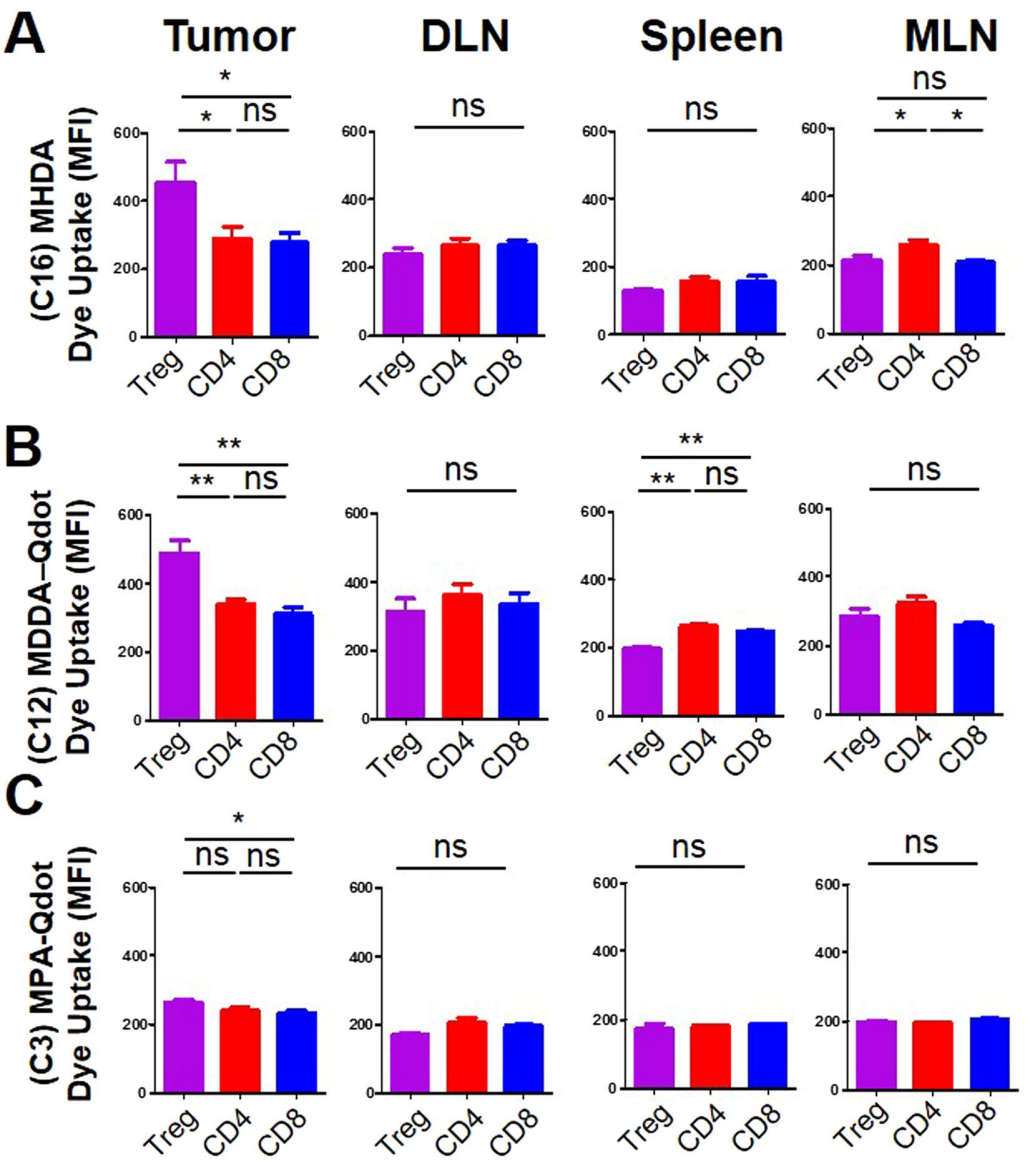
Mice implanted with GL-261 tumors had their brain tumors, Draining lymph nodes (DLN), Spleens, and Mesenteric lymph nodes (MLN) isolated 2 weeks after tumor implantation; FA uptake was determined via flow cytometry. In (**A**), the uptake of MHDA (C16) appended to Qdot-605 across T-cell subsets in different anatomical locations. In (**B**), the uptake of MDDA (C12) appended to Qdot-605 across T cell subsets in different anatomical locations. In (**C**), the uptake of MPA (C3) appended to Qdot-605 across T cell subsets in different anatomical locations. Results are compiled from two separate experiments with 4-5 mice analyzed per group. Statistics are calculated as the percentage of positive population ± SEM. One-way ANOVA, followed by Tukey’s post-hoc analysis, was used to calculate significance. p<0.05*; p<0.01**; p<0.001***. ns = not significant.

We next established intracranial tumors in mice with GL-261, a murine astrocytoma line. After one week of tumor growth, we isolated T cell populations within the brain, draining lymph node (DLN), mesenteric lymph node (MLN), and spleen and stained the cells with FA-Qdot conjugates for 3 minutes using the procedure described above. Through flow cytometic analysis of the active CD4+ populations via Foxp3+, CD62L−, CD44+, we analyzed the differences of three different FA lengths, FA-Qdot (MHDA, C16-Qdot), 3-mercaptopropionic acid (MPA, C3-Qdot) and 16-mercaptododecanoic acid (MDDA, C12-Qdot). We next asked whether fatty acid uptake was different between T cells isolated from the brain, MLN, or spleen. In Fig. 4A, C16-Qdot had statistically different uptake in the MLN, with the Treg subset having the most uptake, as compared to CD8+ and CD4+ populations. In addition, we observe nonspecific uptake between the CD8+, CD4+, and Treg subsets within the brain, DLN, and spleen. This is in direct contrast to the C12-Qdot and C3-Qdot uptake profiles. With the C16-Qdot population, there is specific uptake within the brain tumor microenvironment, which is supported by the literature. The C12-Qdot sample also shows similar levels of uptake, Fig. 4B, as compared to the C16-Qdot sample. In addition, there were no differences in C3-Qdot uptake were observed in the Treg population between the CD4+ and CD8+ population (Fig. 4C **)**. It is likely that the differences between the longer FA chains, C12-Qdot and C16-Qdot, would manifest over a longer period of time, but would not necessarily reflect the initial metabolic effect seen in these small populations. These data demonstrate that activated cells are more likely to utilize FA uptake regardless of cellular subset, and we can determine that, as a population, Tregs more significantly take up FA, as compared to CD4+ effectors and activated CD8+ cells.

### Determination of lymphocyte FA metabolism

**Figure 5:**
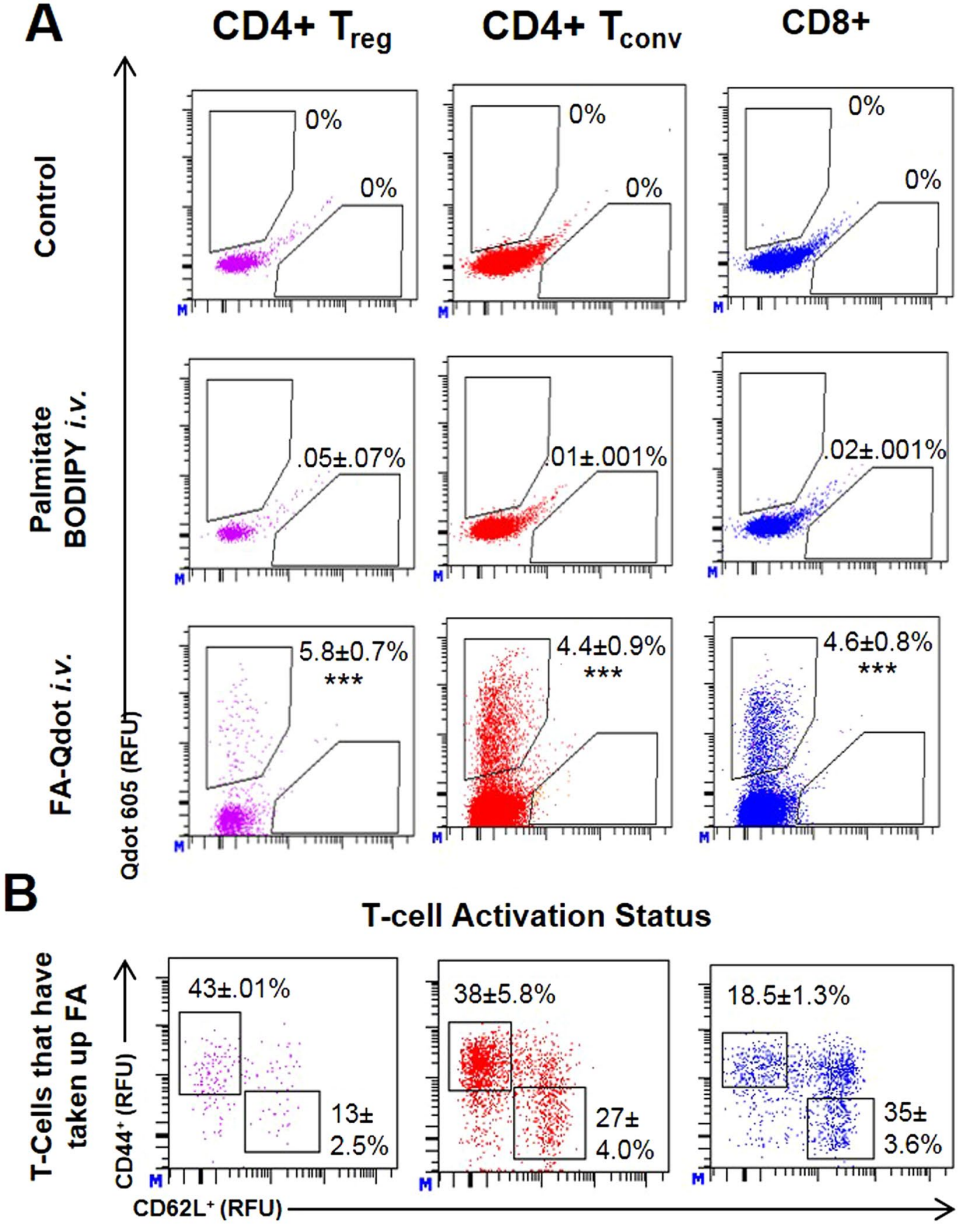
In-vivo uptake of Fatty Acids can be detected by FA-Qdot conjugate, but not by conventional palmitic acid (PA)-BODIPY dye. Wild-type C57/Bl6 mice were injected with 10nM of either FA-Qdot 605 or PA-BODIPY conjugate. After 4 hours, T cells were isolated from the spleen and analyzed via flow cytometry. In (**A**), comparison of particle uptake shown as a percentage of each T cell subset as analyzed via flow cytometry; top panel (NH2) dye, middle panel (PA-BODIPY), bottom panel (FA-Qdot). In (**B**), panels showing the gating strategy for delineating naïve (CD62L+ CD44−) compared to activated T cells (CD62L− CD44+), as both a total of each T cell subset (top panel) and gated only on those that have taken up FA (bottom panel). Results are compiled from four animals. Statistics are calculated as the percentage of positive population ± SEM. One-way ANOVA, followed by Tukey’s post-hoc analysis, was used to calculate significance. p<0.05*; p<0.01**; p<0.001***. Relative Fluorescence Units = (RFU).

To overcome potential problem with *ex-vivo* analysis, that may influence their metabolic properties, we sought to measure fatty-acid uptake *in-vivo*. To verify that the FA-Qdot is a useful sensor for determining FA uptake for T cells obtained from the spleen, we intravenously injected mice with either 2.5 µM FA-Qdot or PA-BODIPY conjugate and analyzed Treg, T-effectors, and CD8+ populations 4 hours later. As demonstrated in Fig. 5A, FA-Qdot conjugates were sensitive enough to detect FA uptake by T cell subsets in the spleen, whereas the PA-BODIPY conjugate was not detected within any of the T cell populations. Furthermore, within the FA-Qdot group, Treg cells had the highest amount of FA uptake, with 5.8% ± 0.7%, compared to 4.4% ± and 4.6% ± 0.8% with the CD4+ T-effector or the CD8+ populations, respectively. We next asked whether there are differences between activated Tregs (CD44+, CD62L−) compared to naïve cells (CD44−, CD62L+) within the spleen, as shown in Fig. 5B. When we further gate on CD44+ and CD62L−, we are able to determine that CD4+ Treg-activated cells had over 3x more uptake, 43% ± 0.01%, as compared to the naïve population, 13% ± 2.5%. Similarly, CD4+ T-conventional populations also demonstrate an increase in FA uptake between the activated and naïve populations: 38% ± 5.8% and 27% ± 4.0%, respectively, although the differences between these populations are not as striking. Interestingly, the levels of intracellular FA were lower in activated CD8+ T cells compared to naïve CD8+ T cells, 18.5% ± 1.3% and 35% ± 3.6%, respectively. To determine if we can measure FA uptake within the tumor, mice were injected intracranially 1 week after tumor implantation with FA-Qdots, for 4 hours and analyzed. As shown in Supplemental Fig. 4 we can distinguish different uptake between the different subsets of the T cells, whereas CD4+ Treg had the most uptake with 20.2% ± 1.1%, CD4+ Tconv had 3.1% ± 0.2% and CD8+ had 5.6% ± 0.2%. These data demonstrate that we are able to detect small changes in fatty acid uptake and that our results are in support of the literature that Treg cells proportionally utilize more FA than other T cells subsets. Intravenous administration of FA did not result in measureable uptake within GL-261 tumor-bearing mice within the 4-hr timeframe. Importantly, this is different from our *ex-vivo* analysis, which showed a trend towards naïve cells taking up FA. This suggests that *in-vivo* labeling of T cells is a more reliable method to determine FA uptake by T cells.

### Establishing metabolic profile preferences in T cell populations

**Figure 6:**
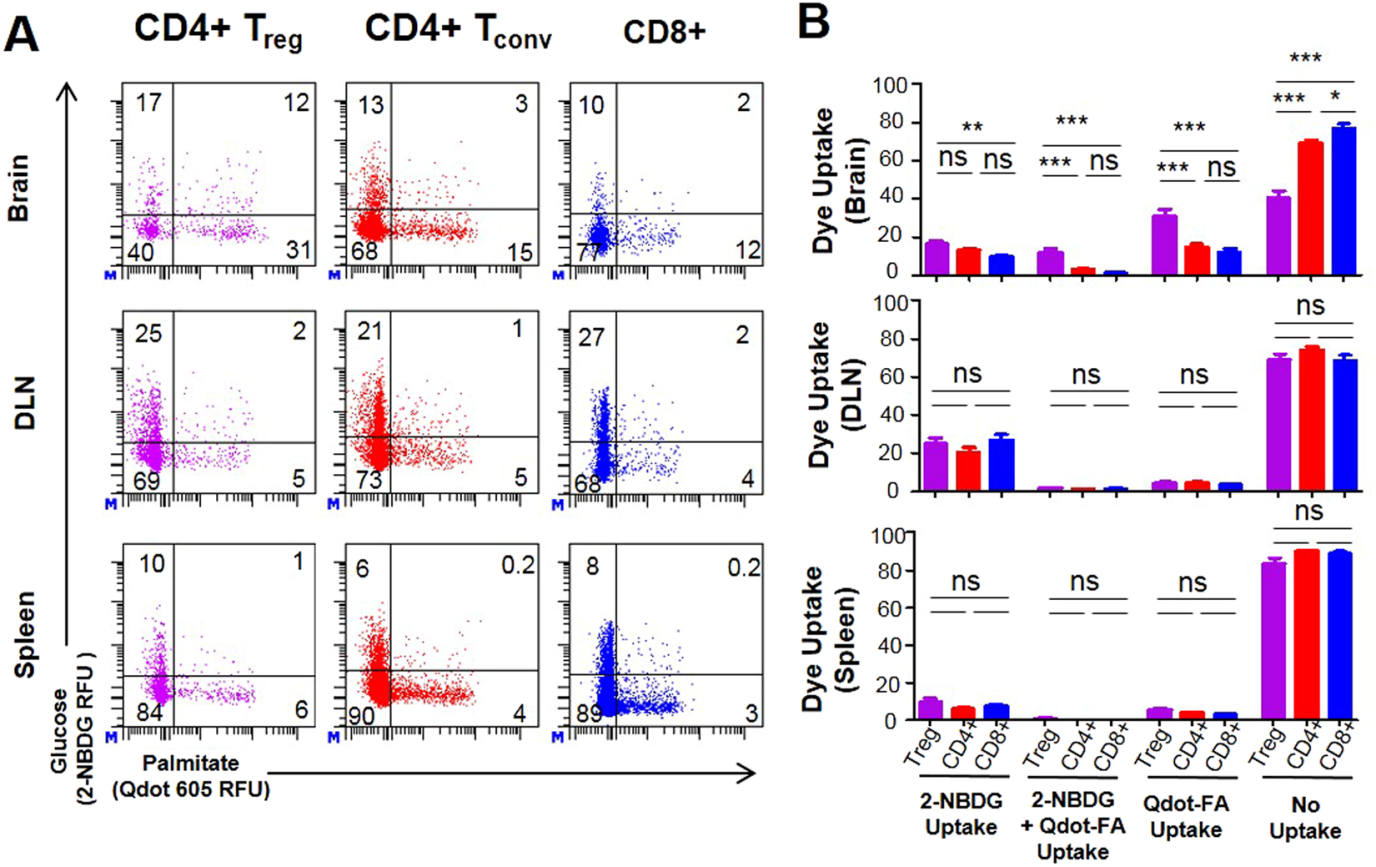
Ex-vivo analysis of both FA-Qdot and Glucose Uptake of T-cells from glioma-bearing mice. Wild-type C57/Bl6 mice were implanted with 4x10^5 GL-261 astrocytoma cells, and after two weeks of tumor growth, T cells were isolated and analyzed via flow cytometry. In (**A**) CD4+CD25+, CD4+CD25−, and CD8+ T cells were assessed for FA-Qdot, and 2-NBDG (fluorescent glucose analog) uptake was examined using ex-vivo labeling for flow cytometry. In (**B**), the comparison of dye uptake across different T cell subsets; in (**C**), an in-depth comparison of fatty acid, 2-NBDG, or simultaneous uptake compared across different tissues in each subset measured. Results are compiled from 5 animals and is representative of 2 experiments. Statistics are calculated as the percentage of positive population ± SEM. One-way ANOVA, followed by Tukey’s post-hoc analysis, was used to calculate significance. p<0.05*; p<0.01**; p<0.001***. RFU = Relative Fluorescence Units; ns= not significant.

Next, we wanted to determine if we could simultaneously analyze glucose and fatty acid uptake within T cell populations. To measure glucose, we chose to use the standard dye 2-NBDG, a fluorescently-labeled glucose analog which can be used in live cell measurements. We established intracranial tumors in mice with GL-261, and after one week of tumor growth, we isolated T cell populations and stained with 2-NBDG and FA-Qdot Fig. 6. The *ex-vivo* T cell analysis shows that a larger percentage of Tregs uptake fatty acid compared to other T cell subsets: 31% ± 2.0% in Tregs versus 15% ± 1.6% and 12% ± 1.8% for CD4 Effectors and CD8+, respectively, Fig. 6A. In comparing the brain, DLN, and spleen, we determined that the CD4+ CD25+ populations have more fatty acid uptake within the tumor microenvironment: 31% ± 2.0%, DLN: 5% ± 0.5% and spleen: 6% ± 0.6%. The CD4+ CD25− and the CD8+ populations had significant differences in the DLN, with 21% ± 2.1% and 27% ± 2.9% of the cells preferentially using 2-NBDG, as compared to 6% ± 0.6% and 8% ± 0.6% in the spleen. This data suggests that there are larger differences in the metabolic profiles in the Treg population within the tumor microenvironment, compared to anatomical locations outside of the tumor, Fig. 6B. Comparison of the data separated by T cell subset can be viewed in Supplemental Fig. 5. Both CD4+ and CD8+ populations both preferentially uptake 2-NBDG, over FA-Qdot *in-vitro* as shown in Supplemental Fig. 6. Furthermore, we observe that proliferating cells are more metabolically active. The trends observed Fig. 6 with CD4+ and CD8+ cells indicate that there is a slight shift favoring glucose metabolism over fatty acid metabolism. This demonstrates that there are significant metabolic differences of T cell subsets demonstrated within the tumor microenvironment.

## Discussion

We have described a new method for determining fatty acid uptake of distinct T cell populations *in-vivo*. Our versatile ligand exchange strategy can accommodate a variety of different fatty acid assays of different chain lengths and emission spectra for ease of analysis. In the context of this manuscript, we were able to measure cellular fatty acid levels of a relatively small cellular population under three different conditions (cultured cells, *ex-vivo* labeling, and *in-vivo)*. Our strategy provides a level of sensitivity that is not easily achieved without isotope labeling. Furthermore, our high level of sensitivity allows for the direct comparison of different cellular conditions in context of metabolic uptake.

The use of quantum dots for *in-vivo* tracking is a popular alternative to fluorescent dye techniques, as they are photo-stable and have narrow emission lines. Our FA-Qdot system can reliably measure the metabolism of fatty acids in immune cells using flow cytometric techniques. Although there are several techniques available to study FA uptake, such as FA-BODIPY conjugate staining, ^13^C-glucose, and MRI studies, they all have significant pitfalls. Measurement of BODIPY is a widespread technique that was first characterized in 2007 by Thumser and Storch in Caco-2 cells^16^. Since its discovery, this fluorescent analog has been used to study FA uptake in a variety of cells and tissues^24^. Unfortunately, the inefficient real time *in-vivo* labeling of this dye conjugate severely limits its applicability to animal studies^22^. Another method uses ^13^C-glucose in media and animal diets to track carbon use in the cells of interest. Unfortunately, this technique requires lipid extraction techniques, followed by MS analysis, to determine FA content^25^. The use of MRI has provided researchers with the resource to look at FA oxidation *in-vivo*; however, this method is both expensive and cannot differentiate between cell populations within the tumor microenvironment. Furthermore, identification of Tregs heavily relies on the identification of Foxp3-expressing CD4+ T cells within *in-vivo* mouse models. A number of Foxp3 reporter models have been engineered that typically express a fluorescent tag, like GFP, YFP, and RFP, when Foxp3+ is expressed^26, 27^. Additionally, models in which Cre recombinase is Foxp3 promoter-driven that involve the conditional knockout of genes only have YFP and GFP as fluorescent reporters. These models interfere with *in-vivo* and *in-vitro* assays of metabolic uptake due to spectral interference with BODIPY, and being able to analyze Treg metabolism via flow cytometry would therefore be incompatible with YFP or GFP fluorescent reporters. Thus, flexibility in color alternatives allows us to address the relative contribution of fatty acid uptake versus glucose uptake by T cells, myeloid cells, and tumor cells simultaneously via flow cytometry or other fluorescence-based assays.

When we explored *ex-vivo* analysis of the distinct T cell populations, we observed differences between short and long chain fatty acids in terms of FA-Qdot staining. Compared to long-chain fatty acids, short chain fatty acids are utilized differently for cellular uptake and do not have active transporters. This may explain the uniform uptake of shorter chain FA in the brains of glioma-bearing animals. Conversely, in our analysis of longer chain FA in within different lymph nodes in comparison to the glioma microenvironment, we observed specific uptake of FA within the Treg population in the brain. These results are further understood by examining the CNS and nutrient availability within the tumor. Starting in the 1960s, a number of studies have addressed the composition of fatty acids within the CNS and within different histological grades of brain tumors^28, 29^. The most abundant non-esterified and non-phospholipid fatty acids are palmitic (saturated) and oleic acid (mono-unsaturated). This abundance of free FA is important for glioma growth, as demonstrated recently in a manuscript by Lin *et al*.^30^, who showed that the oxidation of fatty-acids is important for glioma growth. Additionally, FA uptake appears to be preferentially used by Tregs, which is in consistent with previous reports of Treg metabolic phenotype^14, 15^. Interestingly, myeloid-derived suppressor cells are also wired for this source of energy^31^, which, along with Tregs, accumulate preferentially within the GBM microenvironment. As we are able to determine that Tregs uptake FA more effectively than effector T cell subsets, this suggests that FA uptake is a metabolic program that may contribute to the immunosuppressive environment. We do not see these specific differences in FA uptake in other anatomical locations, such as the DLN and spleen, suggesting that the tumor environment is placing specific metabolic demands on the infiltrating immune cells. This suggests that the metabolic axis of FA uptake and oxidation is the method by which immunosuppression is enforced within the tumor.

Importantly, our approach also allows for the dual measure of fatty acid versus glucose uptake *in-vivo*, which has been previously difficult because the fluorescent analog of glucose, 2-NBDG, has nearly identical spectral characteristics to FA-BODIPY conjugates^17^. Conjugating this assay with 2-NBDG uptake allows us to make stronger observations about the FA environment as compared to glycolysis within tumor bearing mice. Recent studies have shown that the glycolysis of tumors outcompetes T cells for glucose uptake, inhibiting their functionality; the inhibition of glucose uptake by tumors strongly enhances anti-tumor immune response in melanoma^6, 32^. Our data suggests this is happening in the glioma microenvironment, further enhancing immunosuppression in the tumor microenvironment. Interestingly, within the tumor microenvironment, we continually demonstrate a metabolic shift to the use of fatty acids over glycolysis pathways in Tregs. Moreover, we demonstrate that glucose and FA uptake are preferred by distinct T cell populations in the brain and in the periphery, even within each respective immune subset. This suggests a heterogeneity in the metabolic phenotype of immune cells in the body, and in the future, it will be critical to understand the functional difference in these subsets.

In conclusion, we have described a new method for determining fatty acid uptake of distinct T cell populations *in-vivo*. Our ligand exchange strategy is easy to manipulate due to a variety of fatty acid assays of different chain lengths and emission spectra for ease of analysis. In the context of this manuscript, we are able to accurately measure FA uptake in different T cell populations which demonstrates that this technique can be applied to other small cell subsets. Most significantly, we are able to measure FA uptake in three different conditions: cultured cells, *ex-vivo* labeling, and *in-vivo* labeling. In agreement with current literature, we determine that FA uptake is correlated to T cell proliferation^25^, as we have shown in the context of proliferating cells *in-vitro*. The inhibition of FA oxidation and enhancement of glycolysis by T cell subsets might provide the proper environment to reject tumors. Therefore, the ability of our FA-Qdot conjugate to allow for the simultaneous measure of glucose and FA uptake will be critical in understanding how therapeutics may influence the metabolic landscape of the TME.

## Methods

### Fatty acid-conjugated quantum dots

Quantum dots were purchased from Sigma (#748080). 16-Mercaptohexadecanoic Acid (MHDA) (C_16_H_32_O_2_S), 12-Mercaptododecanoic Acid (MDDA), (C_12_H_24_O_2_S), and 3-Mercaptopropionic Acid (MPA) (C_3_H_6_O_2_S) were purchased from Sigma Aldrich. NH2-PEG-SH was purchased from Laysan Bio. Quantum dots were suspended in chloroform and a 1:1000 molar excess of fatty acid or NH2-PEG-SH to Qdot was added, along with 0.05 mM TCEP. The solution was placed at 60 °C and allowed to react until all the materials were in the aqueous layer. The reaction was isolated with an EtOH precipitation and filtered with 10 K MWCO Amicon Centrifugal Filter Devices (Millipore, Darmstadt, Germany) in order to remove excess FA. At this point the solution will be clear at pH 7.4 in PBS with 0.01% BSA. Once cleaned, the dots should be stored at 4 °C and used within 2 weeks. In excess FA solution, the dots are stable in a (+4 C deg,) for up to 3 months. The conjugates are verified through the free FA-test kit (Abcam, Cambridge, MA), and as they were completely passivated with the FA, they were determined to contain approximately 244 ± 5.9 FA per Qdot. To demonstrate the non-toxicity of the dots, a toxicity assay was performed using 10 nM, 100 nM, and 1000 nM of FA-Qdots. 200 µL of each concentration were intravenously injected into the mice and monitored for 15 days; no change in body weight or behavior was noted in all groups (data not shown).

### Stimulated T cell assay

Splenocytes were harvested into a single cell suspension from Foxp3-IRES-GFP mice. The single cell suspension was then lysed for two minutes at room temperature with ACK lysis buffer. Purified T cell isolation was performed using the EasySep Pan T cell isolation kit following the manufacturer’s protocol (StemCell Technologies, Vancouver, CA). T cells were labeled with Cell Trace Violet (CTV) dye (Invitrogen, Waltham, MA) following the manufacturer’s instructions. After dye labeling, T cells were thoroughly washed and resuspended in complete RPMI (10% FBS, Glutamax, 50 um Beta-mercaptoenthanol) containing 3 ug/ml anti-CD28 and recombinant murine IL-2. 250,000 purified T cells were plated per well in a 96-well plate, which had been previously coated overnight with 2.5 ug/ml of anti-CD3 antibody (Biolegend, Santa Cruz, CA). After 72 hours of proliferation, the T cells were harvested, labeled with a APC-efluor780 viability dye (Ebioscience, San Diego, CA), and stained with the relevant flow cytometry antibodies, lipid dyes, or blocking FA immediately before flow cytometry analysis.

### Flow cytometry analysis

For *in-vitro* studies, the following flow cytometry panel was used in conjunction with CTV and viability dye: anti-CD3 Alexa700, anti-CD4 PE-Cy7, anti-CD8 APC, and anti-CD44 PE, all at a 1:200 dilution (Biolegend). For *in-vivo* and *ex-vivo* studies, the following flow cytometry panel was used: anti-CD3 PE-Cy7, anti-CD4 Pacific Blue, anti-CD8 APC, anti-CD44 PE, anti-CD62L APC-Cy7, and anti-CD25 Alexa 700, all at a 1:200 dilution, except anti-CD25 at a 1:50 dilution (Biolegend). Endogenous Foxp3 expression was detected either via eGFP of eYFP fluorescence, which is compatible with all panels but not when used with BODIPY-FL dye (493/503) or 2-NBDG (465/540). In those assays, Foxp3-based uptake of FA or Glucose was not performed, but all other cellular subsets described were tested. All acquisition was performed using a BD Fortessa flow cytometer (BD, MD, USA) at the Northwestern University Comprehensive Flow Cytometry core.

### FA-Dye conjugate labeling

For *in-vitro* and *ex-vivo* assays, fatty acid uptake was performed as previously described^25^. After antibody staining, T cells were washed and suspended in 50 ul of room temperature PBS. 50 ul of 2X concentrated labeled-FA (either FA-Qdot or FA-BODIPY) were added to the wells and incubated for 3 minutes. Immediately after incubation, 200 ul of ice-cold PBS was added to stop FA uptake. Cells were then washed two times and taken directly to flow cytometry analysis. This method has been previously reported for the optimal measurement of FA uptake^22^. It is important to note that the FA-dye uptake assay occurs 72 hrs after CTV labeling and T cell proliferation, allowing us to ascertain the uptake of individual T cells at different cycles of proliferation. For FA-uptake blocking assays, T cells cultures or *ex-vivo* cell suspensions are pre-incubated with a 1 mM of Palmitic acid for 10 min at room temperature before the addition of labeled FA.

For *in-vivo* analysis, FA conjugates were injected either *i*.*v*. or directly into the intracranial cavity (i.c.) at the concentrations described for each experiment. For experiments involving tumor-bearing mice, the injections were performed at 1 week post-tumor implantation. Four hours after FA-conjugate injections, peripheral organs were immediately harvested separately into single cell suspension, stained with flow cytometry antibodies, and analyzed using the BD Fortessa flow cytometer. Brain tissue was harvested into single cell suspension, re-suspended in a 30% Percoll gradient solution, and overlaid on 70% Percoll (GE Healthcare, Pittsburg, USA). The gradient was then spun at 1200 x g for 30 min at 4 °C, and the interface of the gradient was obtained, which contained isolated leukocytes from the brain tumor tissue as described previously^33^. This was taken forward for flow cytometry analysis.

## Electronic supplementary material

Supporting Information
