# Supplementary material for: Fatty Acid Uptake in T Cell Subsets Using a Quantum Dot Fatty Acid Conjugate
Source: Sci Rep. 2017 Jul 19;7:5790. doi: 10.1038/s41598-017-05556-x (PMC5517517; doi:10.1038/s41598-017-05556-x)
Supplement: Supplementary file 1 — Supporting Information [file 41598_2017_5556_MOESM1_ESM.pdf]

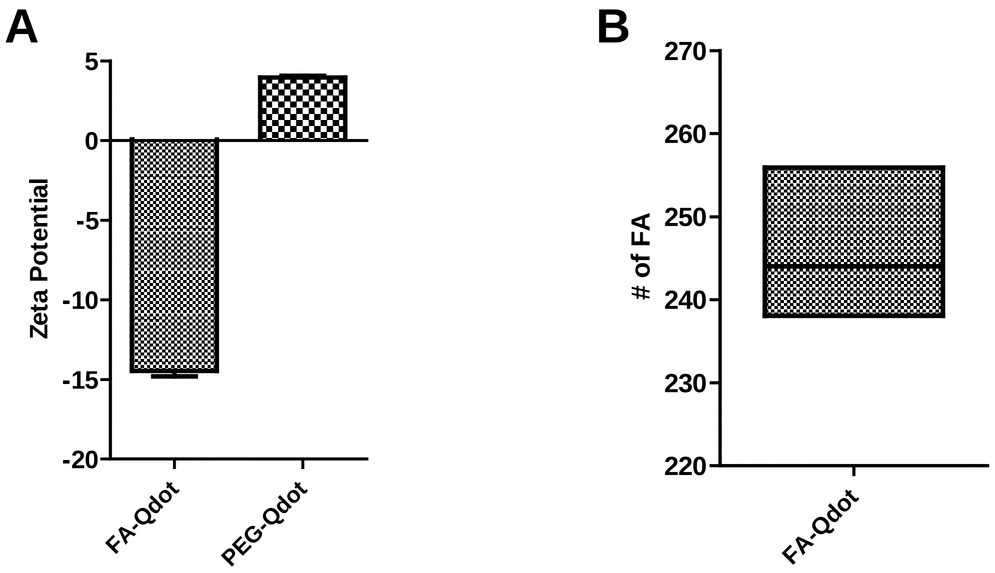

Figure S1. Characterization of FA-Qdot. A) The zeta potential of FA-Qdot and PEG-Qdot. Triplicate measurements determined the zeta potential to be -14.47 for FA-Qdot and +3.94 for PEG-Qdot. B) Free fatty acid test to determine the amount of FA present at the surface of the Qdot. We determined an average of 244 ± 5.9 FA ligands per Qdot, measured in triplicates. To account for any potential background noise from Qdot absorption, we subtracted the average of the PEG-Qdot from the values measured for the FA-Qdot.

Figure S1.

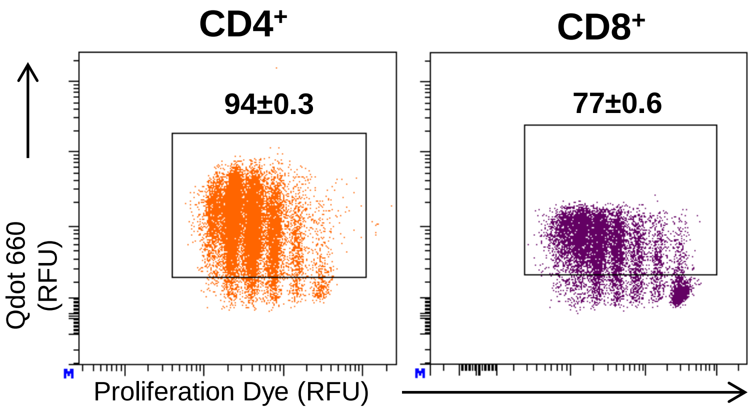

Figure S2. Qdot 660 appended to palmitic acid also shows T cell uptake *in-vitro*. Cell Trace Violet (CTV)-labeled T cells were cultured under stimulating conditions of anti-CD3/anti-CD28 treatment for 72 hours and assayed *in-vitro* for Qdot 660-FA uptake via flow cytometric analysis. Statistics are shown as the percentage of positive population ± SEM. RFU = Relative Fluorescence Units**.**

Figure S2.

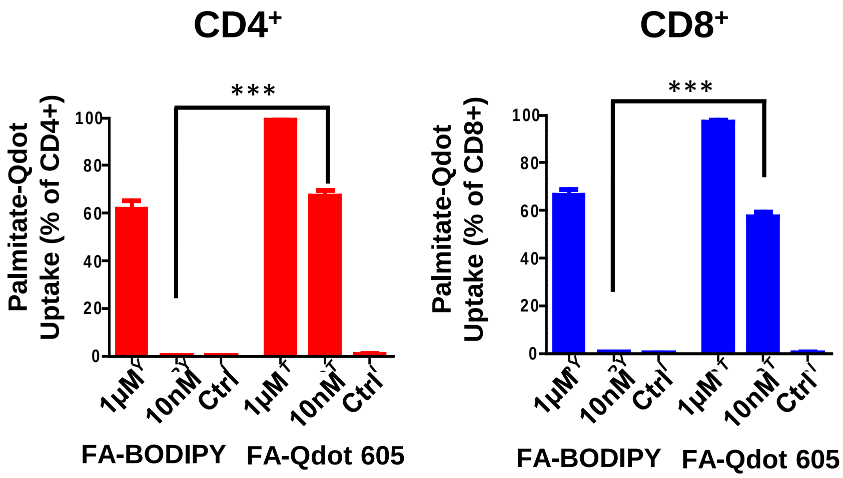

Figure S3. Comparison of FA-BIDOPY to FA-QDOT 605 sensitivity uptake by T cells *in-vitro*. Cell Trace Violet (CTV)-labeled T cells were cultured under stimulating conditions of anti-CD3/anti-CD28 treatment for 72 hours and assayed for FA uptake at different concentrations via flow cytometric analysis. The percentage of T cells up taking FA are shown at each concentration listed above. Statistics are calculated as the percentage of positive population ± SEM. One-way ANOVA, followed by Tukey's post-hoc analysis, was used to calculate significance. $p<0.05$*; $p<0.01$**; $p<0.001$***.

Figure S3.

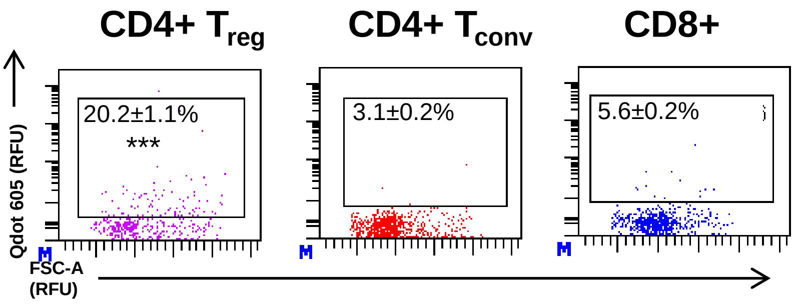

Figure S4. Uptake of Qdot-Palmitate *in-vivo* within Brain Tumors. Wild-type C57/Bl6 mice were injected with nM of either Palmitic acid- Qdot 605 and after 4 hours, T cells were isolated from the spleen and analyzed via flow cytometry. In (C), the particle uptake determined after intracranial administration shown as a percentage of each T cell subset, as analyzed via flow cytometry. Results are compiled from four animals. Statistics calculated as the percentage of positive population ± SEM. One-way ANOVA, followed by Tukey's post-hoc analysis, was used to calculate significance. $p<0.05$*; $p<0.01$**; $p<0.001$***. Relative Fluorescence Units = (RFU).

Figure S4.

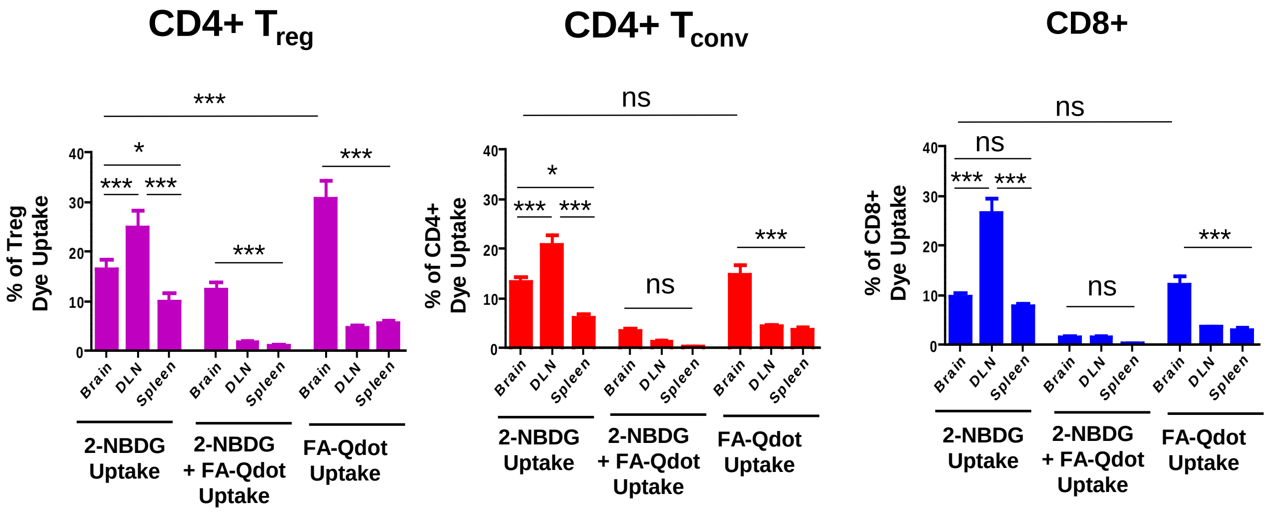

Figure S5. ***Ex-vivo*** **analysis of both FA-Qdot and Glucose (2-NBDG) Uptake of T-cell from glioma-bearing mice. Wild-type C57/Bl6 mice were implanted with 4x10^5 GL-261 astrocytoma cells, and after two weeks of tumor growth, T cells were isolated and analyzed via flow cytometry. In (C), an in-depth comparison of FA-Qdot, 2-NBDG, or simultaneous uptake across different tissues in each subset measured. Results are compiled from 5 animals and is representative of 2 experiments. Statistics are calculated as the percentage of positive population ± SEM. One-way ANOVA, followed by Tukey's post-hoc analysis, was used to calculate significance. p<0.05*; p<0.01**; p<0.001***. ns= not significant.**

Figure S5.

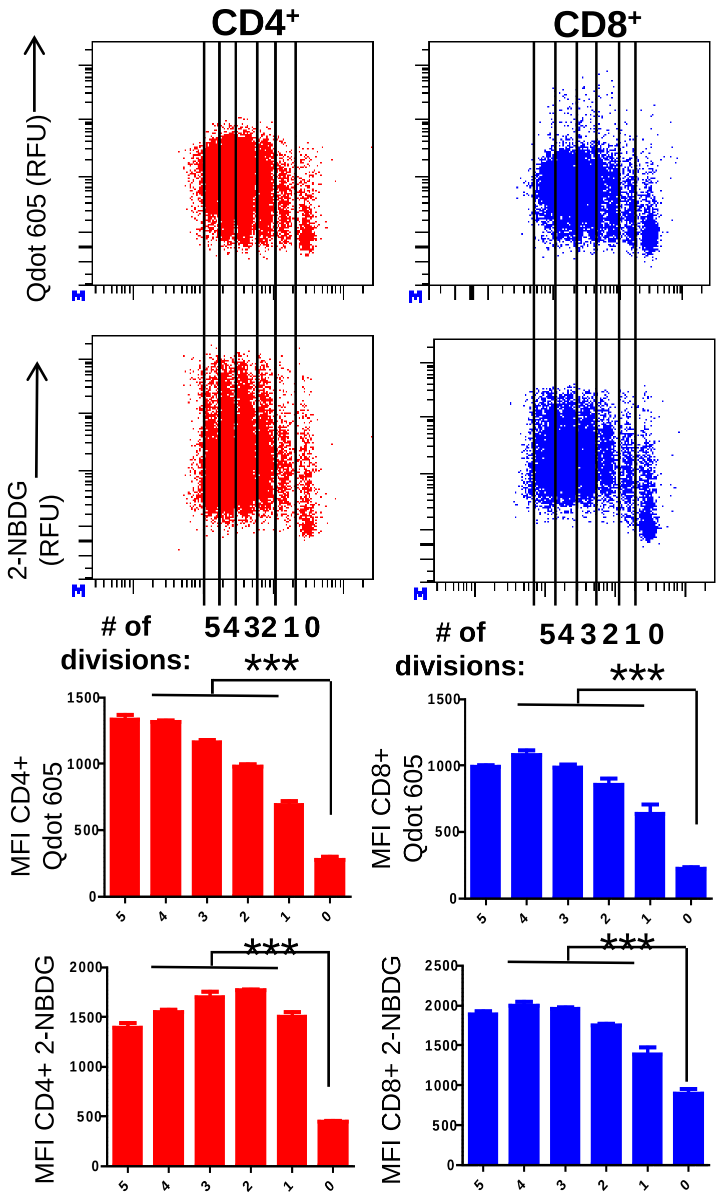

Figure S6. Fatty Acid (FA-Qdot) and glucose (2-NBDG) uptake determined by lymphocytes during in-vitro culture. Cell Trace Violet (CTV)-labeled T cells were cultured under stimulating conditions of anti-CD3/anti-CD28 treatment for 72 hours and assayed for FA and 2-NBDG uptake utilizing flow cytometric analysis. Stimulated T cells were stained for T cell markers and incubated with 10nM of FA-Qdot 605 conjugate for 3 minutes before running flow analysis. Results are compiled from four separate experimental wells per group and is representative of 2 experiments. Statistics are calculated as the percentage of positive population ± SEM. One-way ANOVA, followed by Tukey's post-hoc analysis, was used to calculate significance. p<0.05*; p<0.01; [illegible]**

Figure S6.
